# Signs of Lesions of Nerve Centers

**Published:** 1858-12

**Authors:** C. B. Chapman


### SIGNS OF LESIONS OF NERVE CENTERS.

BY PROF. C. B. CHAPMAN.

WE have already described the offices performed by the different nerve centers, which has suggested the fact that there are four distinct functions contributed by these axes, as they exist in the higher animals and in man, besides those which are furnished through the agency of the nerves of special sense, or those which contribute to the functions of *vision*, *olfaction*, and *audition*, each of which seems to be independent and distinct in itself. Beginning, therefore, in the animal scale, we find the excito-motor, or spinal system, independent in the origin of each nerve for these separate functions of sensation and of motion, but united after passing but a few lines from their origin. Lesions of the spinal center will exhibit different phenomena which will accord with their location, whether it be in the anterior or posterior columns, or whether both columns are at the same time involved. Lesions of the spinal centers have been named centric and eccentric. The usual phenomena which may result from such lesions, will be learned by a few carefnl experiments upon living animals, for the functions of the spinal cords are found to correspond, from man down to the lowest vertebrate, the only difference consisting in the amount of control which can be exercised over this by the encephalic centers. If an afferent or sensitive branch of a spinal nerve be divided just before it unites with its efferent fellow, sensation of the part to which such nerve is distributed will be impaired or lost, but the faculty of motion may still exist. If instead of the afferent nerve, the efferent has been divided, power of motion will be destroyed, but sensation may preserve its integrity. If, however, the lesion be made just beyond, or at the point of union of the sensitive and motor branch, both functions beyond that point will be destroyed, but the stump of the sensitive branch will still respond to irritants, and the stump of the motor branch will maintain, and may exhibit its functional integrity, provided there be the smallest amount of contractile muscular tissue along its course upon which it can act. Injury or disease of a nerve trunk, far away from its center, may produce perverted sensation, or impaired or perverted muscular action. Injury or disease of the spinal cord, or a limited portion of this center, may produce the same phenomena, although it will be more likely to produce loss of sensation and motion. We are, therefore, in many instances, deprived of such exact measure of knowledge with regard to the sources of these phenomena, whether they be centric or eccentric. The sources of such phenomena will sometimes be so apparent, that they can not be mistaken by the most inattentive observer, while in other cases, their obscurity may be such as to place them beyond the knowledge of the most careful investigator. Much aid may be afforded in such investigations, through a careful knowledge of its antecedents, provided the condition was produced by traumatic injury. A lesion, situated in one of the lateral columns of the spinal cord, will produce loss of motion only if such lesion be confined to the anterior lateral column, but the effect will be loss of sensation if the injury be situated in the posterior lateral. If, however, both columns of one side be involved, there will be loss of sensation and motion of that side, and such condition is called *hemiplegia.* When the upper or lower half of the body is paralyzed, a lesion of the four columns of the cord may be suspected, and the disease is denominated *paraplegia.*

It will often be difficult to distinguish whether irregular nerve action is the result of centric or of eccentric lesion.

If the result be loss of sensation and of motion, the lesion is most commonly in the center—it is centric. If the manifestation be irregular motion, (which constitutes chorea or St. Vitus' dance) the trouble may be at the root, or along the nerve—it is eccentric. These phenomena, however, are by no means uniform, for the most careful investigators are well aware how uncertain and even impossible, with our present knowledge, it is to know the situation of a lesion with entire certainty by its manifestations, although the diligent student may learn wherein there are exceptions to this general rule. The condition of the being may at once afford unmistakable evidence of the origin or cause of irregular action, but the phenomena observed will afford us no certain evidence. Manifestations of centric lesions are commonly more palpable than those of the eccentric kind. We have, however, one well marked exception in that dreadful malady, which results from the injury of a remote nerve, called *tetanus* or lock-jaw. We have in this condition, a well marked example of the *eccentric* or reflex manifestation of nerve power. Such injury will induce that muscular contraction peculiar to this disease, first through the agency of the nerves connected with the posterior columns of the spinal cord. These are the nerves of sensation. The irritation thus transmitted to this center is further conveyed, through the agency of the connecting fibers within the cord, to the anterior or motor column, by the agency of the nerves of which the irritation is reflected back to the point of injury, so that by the combined action of the two systems that phenomenon is produced which is so characteristic of this dreadful malady. In this disease, notwithstanding the injury is remote from the spinal cord, this center, while uninjured itself, becomes an instrument for the most grave manifestations through the further agency of the muscular system.

These are questions which ought to engage the most careful investigations of the naturalist, the physiologist, and the practitioner, in order that they may be enabled to require rules by which he can (approximately at least) determine the location of the cause of these manifestations, and whether they originate in a center or in remote nerves. We have no evidence that lesions of the spinal center ever extend their effects upward to the cerebrum and cerebellum, except so far as they may be effected in common with the whole organism. Not so, however, with regard to the influence of the higher centers upon this, for if the cerebellum be injured, the principal evidence of its lesion will be found in the perverted operations of the spinal system, and this we would infer when we remember that it is the office of this center to preside over the spinal system, in a way that will secure harmony of muscular motion.

Malformations, or injuries of the *cerebellum* or *cerebrum*, may transmit their effects to the spinal system, which may furnish the only evidence in our possession of the existence of such lesion. Such lesions are the common cause of that obscure and thus far undefinable disease, called epilepsy.

